# Gene Expression Analyses of Subchondral Bone in Early Experimental Osteoarthritis by Microarray

**DOI:** 10.1371/journal.pone.0032356

**Published:** 2012-02-27

**Authors:** RongKai Zhang, Hang Fang, YuXian Chen, Jun Shen, HuaDing Lu, Chun Zeng, JianHua Ren, Hua Zeng, ZhiFu Li, ShaoMing Chen, DaoZhang Cai, Qing Zhao

**Affiliations:** 1 Department of Orthopaedics, The Third Affiliated Hospital, Sun Yat-sen University, Guangzhou, China; 2 Department of Orthopaedics, The Fifth Affiliated Hospital, Sun Yat-sen University, Zhuhai, China; Ohio State University, United States of America

## Abstract

Osteoarthritis (OA) is a degenerative joint disease that affects both cartilage and bone. A better understanding of the early molecular changes in subchondral bone may help elucidate the pathogenesis of OA. We used microarray technology to investigate the time course of molecular changes in the subchondral bone in the early stages of experimental osteoarthritis in a rat model. We identified 2,234 differentially expressed (DE) genes at 1 week, 1,944 at 2 weeks and 1,517 at 4 weeks post-surgery. Further analyses of the dysregulated genes indicated that the events underlying subchondral bone remodeling occurred sequentially and in a time-dependent manner at the gene expression level. Some of the identified dysregulated genes that were identified have suspected roles in bone development or remodeling; these genes include Alp, Igf1, Tgf β1, Postn, Mmp3, Tnfsf11, Acp5, Bmp5, Aspn and Ihh. The differences in the expression of these genes were confirmed by real-time PCR, and the results indicated that our microarray data accurately reflected gene expression patterns characteristic of early OA. To validate the results of our microarray analysis at the protein level, immunohistochemistry staining was used to investigate the expression of Mmp3 and Aspn protein in tissue sections. These analyses indicate that Mmp3 protein expression completely matched the results of both the microarray and real-time PCR analyses; however, Aspn protein expression was not observed to differ at any time. In summary, our study demonstrated a simple method of separation of subchondral bone sample from the knee joint of rat, which can effectively avoid bone RNA degradation. These findings also revealed the gene expression profiles of subchondral bone in the rat OA model at multiple time points post-surgery and identified important DE genes with known or suspected roles in bone development or remodeling. These genes may be novel diagnostic markers or therapeutic targets for OA.

## Introduction

Osteoarthritis (OA) is a complex degenerative joint disease that affects millions of middle-aged and older individuals. It is characterized by progressive cartilage erosion, osteophyte formation, subchondral bone modification, and synovial inflammation, which follow alterations in the biomechanical and biochemical properties of the joint [1].

OA is generally considered to be a cartilage disease, but increasing evidence indicates that it is also a bone disease [2]–[5]. Changes in subchondral bone may occur prior to the onset of cartilage degeneration [6]. Subchondral bone consists of the subchondral bone plate and the underlying trabecular bone and bone marrow space [7]. Alterations of the subchondral bone increase with the progression of OA. It has been shown that subchondral bone is an effective shock absorber, and nutrients or cytokines can be transported from the subchondral bone to the overlying cartilage via clefts or channels in the tidemark. Subchondral bone cells influence cartilage metabolism [3]–[5], [8], [9]; however, it is still debated whether changes in subchondral bone precede or follow cartilage destruction [10]. Therefore, it is crucially important to understand the molecular characteristics of subchondral bone changes in vivo, especially during the early stages of OA. This information is important because any identified alterations in this period could contribute to the development of new diagnostic markers or therapeutic targets for OA [11].

With the development of microarray technology, changes in the expression levels of thousands of genes can be examined simultaneously, and integral analysis of the dysregulated genes can be performed to obtain information regarding pathogenic mechanisms of OA at the cellular level. Furthermore, microarray data can be used to discover novel molecular diagnostic markers and therapeutic targets [12]–[15]. A small number of studies have reported the gene expression profiles of articular cartilage and bone from human or animal OA samples. These reports have provided important diagnostic markers and therapeutic targets for OA [16]–[18]; however, gene expression profiles and the chronology of OA-induced changes in subchondral bone that are associated with cartilage degeneration remain poorly understood.

It is currently impossible to obtain adequate subchondral bone samples from humans to study the initiation and early stages of OA. Moreover, studies of ex vivo cultures from osteoarthritic subchondral bone have not precisely identified the molecular changes that occur in vivo [8], [9], [13], [19]. Therefore, it is necessary to examine the early stages of OA in subchondral bone using animal models. By performing medial meniscectomy and medial collateral ligament transection, a surgically induced rat model of OA was used to address the questions posed by this study. This animal model is consistent with post-traumatic OA in humans and can be used for the genetic analysis of OA [20]–[22].

For the first time, we describe a simple and effective method of separation of subchondral bone sample from the knee joint in a rat model of OA, which can effectively avoid bone RNA degradation. This procedure can be used for genetic analyses, and it effectively avoids the RNA degradation that may occur in subchondral bone samples during the operation. In addition, this work is the first study to use microarray technology to elucidate the time course of the molecular changes that occur in the subchondral bone just beneath damaged cartilage in the early stages of experimental OA.

## Results

### Validation of the rat model of osteoarthritis

**Figure 1:**
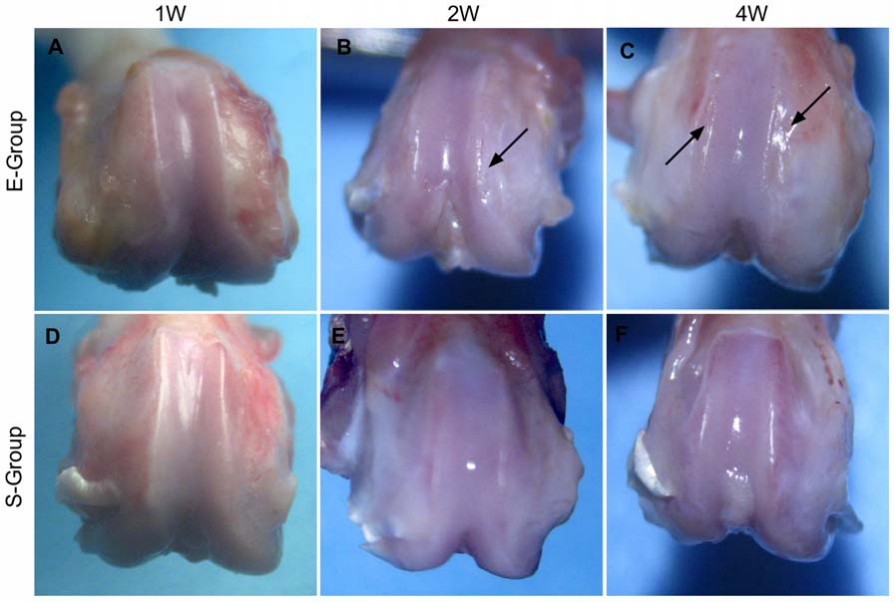
Macroscopic analysis of femoral condyles in a rat model of osteoarthritis. No detectable macroscopic surface changes were observed in the femoral condyle (**A**) of E-group rats at 1 week post-surgery. At 2 weeks post-surgery, the local medial femoral condyle of the distal femur exhibited a slightly rough articular surface (**B**, arrow). Significant roughness of the articular surface was observed both on the medial and lateral femoral condyle (**C**, arrow) at 4 weeks post-surgery. The knee joints (**D**, **E**, **F**) of the S-group exhibited a normal articular surface at all time points following the surgery.

**Figure 2:**
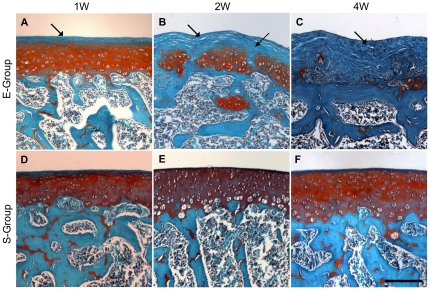
Histologic analysis of cartilage degradation induced by medial meniscectomy and medial collateral ligament (MCL) transection, evaluated by section staining with safranin-O and rapid green. The sections were stained with safranin-O (red stain) for glycosaminoglycans, rapid green for bone and fibrous tissue (green stain), and counterstained with Mayer's hematoxylin for nuclei (blue). A slight decrease in glycosaminoglycan staining at 1 week post-surgery is shown (**A**, arrow). A loss of superficial cartilage and focal fibrillation of the articular surface at 2 weeks post-surgery was seen (**B**, arrow). A focal loss of chondrocytes and exposure of subchondral bone wear observed at 4 weeks post-surgery (**C**, arrow). A healthy articular surface was observed throughout the study (**D**, **E**, **F**). Each of the above images was captured from representative locations from sections of femur condyles and is shown at the same magnification. The scale bar represents 200 µm.

All animals in this study were healthy, as indicated by the maintenance of normal body weight and food consumption; their normal walking patterns returned within five days post-surgery. The rat model of OA was validated by gross morphological and histological analysis. Similar to the disease progression that has been observed in humans, the gross morphological and early histological events associated with OA occurred in a time-dependent manner in the animal model. These findings confirmed that a medial meniscectomy and medial collateral ligament (MCL) transection effectively induced OA-like early changes in the cartilage, as reported previously (**Figure 1** and **Figure 2**) [21], [22].

### Integral analysis of DE genes in the subchondral bone of E-Group versus S-Group samples

**Figure 3:**
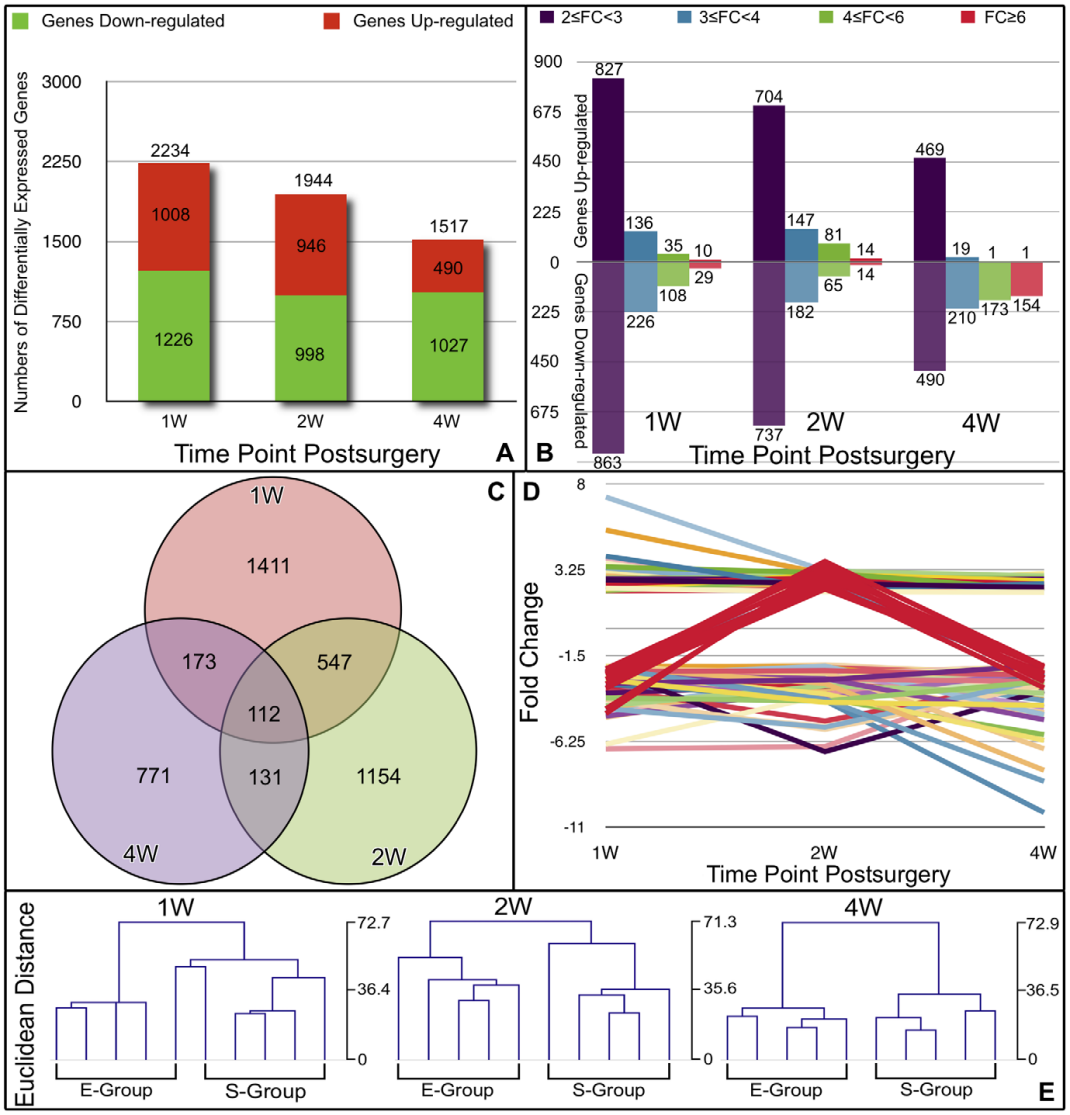
Integrated analyses of the DE genes in the subchondral bone of E-Group versus S-group samples. The number of DE genes at each time point is shown in **A**. DE genes were classified according to their differential expression levels with a minimum of 2-fold, 3-fold, 4-fold and 6-fold differences (**B**). Venn diagram depicting the overlap of dysregulated genes at three time points post-surgery (**C**). The expression patterns of 112 genes that were differentially expressed at all three time points are shown in **D**. Dendrogram of the unsupervised hierarchical clustering analysis of the E-Group and S-group samples at each time point is shown in **E**. The clustering was performed based on DE genes of the E-group versus the S-group at each time point. Euclidean distances were used to measure the similarities between the expression profiles of the samples.

Integral analysis of gene dysregulation could lead to a better understanding of the pathogenic mechanisms of OA. The results of these analyses indicate that the number and type of DE genes in the subchondral bone vary over time. As shown in **Figure 3 A** and **B**, the microarray data analyses identified 2,234 DE genes at 1 week post-surgery, including 1,008 up-regulated genes and 1,226 down-regulated genes. Further analysis revealed 1,690 DE genes with greater than 2-fold expression level changes, 362 genes with changes greater than 3-fold expression level changes, 143 with changes greater than 4-fold expression level changes, and 39 genes with changes greater than 6-fold expression level changes.

At 2 weeks post-surgery, 1,944 DE genes were identified, including 946 up-regulated genes and 988 that were down-regulated. Of the DE genes, 1,441 exhibited a greater than 2-fold change in their expression levels, 329 displayed a greater than 3-fold change, 146 genes displayed a greater than 4-fold change, and 28 displayed a greater than 6-fold change.

Furthermore, 1,517 DE genes were identified at 4 weeks post-surgery, including 490 that were up-regulated and 1,027 that were down-regulated. Of these genes, 959 exhibited a greater than 2-fold change, 229 genes displayed a greater than 3-fold change, 174 genes displayed a greater than 4-fold change, and 155 genes displayed a greater than 6-fold change.

Specifically, the numbers of up-regulated and down-regulated genes were similar at 1 and 2 weeks post-surgery. At 4 weeks, however, a large number of DE genes with down-regulated expression (approximately 67.7%) were identified, indicating that during late-stage OA, subchondral bone cells had a more limited potential to generate the functional mediators involved in bone tissue homeostasis than did subchondral bone cells in early stage OA.

Interestingly, the DE genes that exhibited differential regulation at two of the three time points appeared to be dysregulated in a similar manner. Of the 659 genes that were dysregulated at both the 1- and 2-week time points (**Figure 3C**), 86.6% were dysregulated in the same direction (i.e., 30.6% were up-regulated at both time points, and 56.0% were down-regulated), and 13.4% were down-regulated at 1 week and up-regulated at 2 weeks. Similarly, of the 243 genes that were differentially expressed at 2 and 4 weeks, 90.5% were dysregulated in the same direction (i.e., 37.9% were up-regulated at both time points, and 52.6% were down-regulated; 9.5% were up-regulated at 2 weeks and down-regulated at 4 weeks).

Only 112 genes were differentially expressed at all three time points (**Figure 3D**). Of these genes, 101 genes were dysregulated in the same direction (41 were up-regulated, and 60 were down-regulated), Eleven were down-regulated at 1 week, up-regulated at 2 weeks, and down-regulated at 4 weeks. Detailed information on these genes can be found online in **Supplementary Table S1**. The interesting pattern of uniform dysregulation of the DE genes may be indicative of the molecular changes occurring in the subchondral bone. Alternatively, this finding may have been due to false positive results in the microarray data or in the subsequent analyses.

Furthermore, of the 112 genes that were differentially regulated at all three time points, 58% were poorly annotated, i.e., the functions of these genes and the biological processes in which they are involved are unclear. Of the annotated genes that were differentially expressed at all time points, 23 were consistently up-regulated and 22 were consistently down-regulated. Two genes were down-regulated at 1 week, up-regulated at 2 weeks, and down-regulated at 4 weeks. There was no clear relationship between these dysregulated genes. However, several genes that may play pivotal roles in OA are listed in **Supplementary** **Table S2**. Almost none of these genes have been described in the context of OA, with the exception of Ednra, which mediates endothelin 1 signaling and has been considered a potential drug target for reducing OA pain [23].

### Unsupervised hierarchical clustering of sample probes

Unsupervised hierarchical clustering was performed based on the target values of the DE genes from the E-Group versus the S-Group at each post-surgical time point. The Euclidean distance was used to measure the degree of similarity between the expression profiles of the samples. As expected, clustering of the results at each time point resulted in E-Group sample clusters and S-Group sample clusters, although one of the E-Group samples clustered closer to the S-Group samples at the 1-week time point. The dendrogram in **Figure 3E** shows the relationships between the expression levels of the samples and suggests that the E-Group samples' gene expression profiles were distinct from the S-group samples at each time point. These data further demonstrate that subchondral bone changes at the gene expression level occurred in a time-dependent manner in OA pathogenesis.

### Analysis of DE genes with known or suspected roles in bone development or remodeling

Gene function analysis revealed a number of DE genes that have known or suspected roles in bone development or remodeling. Genes with known roles in osteoclast differentiation and function include Acp5, Ctsk, Csf1, Ihh, Ostf1, Mmps (Mmp1b, 2, 3, 12, 13, 14, 16, 23, 24), Tnfsf11/RANKL, and Tnfrsf11b/OPG (**Table 1**). The genes involved in osteoblast differentiation and function include Alp, Angptl2, Angptl1, Cbfβ, Col-1α1, Col-1α2, Col-3α1, Col-4α1, Mapk6, Igf1, Igfals, Igfbp1, Osr2, Postn, Pth, Pthlh, and Pth1r (**Table 2**). A subset of the genes is known to be related to OA including Adamts5, Aspn, Bmp1, Bmp3, Bmp4, Bmp5, Bmpr1a, Cald1, Col-2a1, Col-5a1, Col-5a2, Col-5a3, Col-6a2, Col-8a1, Col-9a1, Col-9a2, Col-9a3, Col-11a2, Col-16a1, Col-18a1, Col-27a1, Ctnnβ1, Ddr2, Fgfr2, Fgfr3, Fgfbp3, Fap, Fgf13, Gbx2, Mst1, Igsf10, Sdc2, Sfrp2, Tgfβ1, Wisp1, and Wisp3. Most of the genes in the last group were primarily up-regulated at 1 week post-surgery (**Table 3**). These results indicated that an altered phenotype of subchondral osteoclasts and osteoblasts may be contributing factors in OA.

**Table 1:** Representative DE genes with known or suspected roles in osteoclast differentiation and function [11], [32]–[34], [36], [38].

			Fold-change		
Genebank	Symbol	Description	1W	2W	4W
NM_019144	Acp5	Acid phosphatase 5, tartrate resistant	2.42**		
NM_031560	Ctsk	Cathepsin K			4.18**
NM_023981	Csf1	Colony stimulating factor 1 (macrophage)			−4.39**
NM_053384	Ihh	Indian hedgehog		3.41**	2.32**
NM_148892	Ostf1	Osteoclast stimulating factor 1			−4.37**
XM_001072313	Mmp1b	Matrix metalloproteinase 1b (interstitial collagenase)		3.39**	
NM_031054	Mmp2	Matrix metallopeptidase 2	3.74**		
NM_133523	Mmp3	Matrix metallopeptidase 3	6.62**	5.51**	
NM_053963	Mmp12	Matrix metallopeptidase 12	4.26*		
NM_133530	Mmp 13	Matrix metallopeptidase 13	2.83**		
NM_031056	Mmp 14	Matrix metallopeptidase 14	2.61*		
NM_080776	Mmp 16	Matrix metallopeptidase 16	2.12*		
NM_053606	Mmp23	Matrix metallopeptidase 23	2.14*		
NM_031757	Mmp24	Matrix metallopeptidase 24			2.31**
TC609946	TIMP1	Metalloproteinase inhibitor 1 precursor, partial (70%)	2.52**		
NM_057149	Tnfsf11(RANKL)	Tumor necrosis factor (ligand) superfamily, member 11	2.81**		
NM_012870	Tnfrsf11b (OPG)	Tumor necrosis factor receptor superfamily, member 11b		2.12*	

* = *P*<0.05,

** = *P*<0.01, - = down-regulated (E-Group versus S-Group samples).

**Table 2 pone-0032356-t002:** Representative DE genes with known or suspected roles in osteoblast differentiation and function [3], [5], [6], [11], [14], [16], [19].

			Fold-change		
Genebank	Symbol	Description	1W	2W	4W
NM_013059	Alp	Alkaline phosphatase,liver/bone/kidney	−2.93**		−3.55**
NM_133569	Angptl2	angiopoietin-like 2	2.19**		
NP_001102853	Angptl1	angiopoietin-like 1		4.57**	2.63**
NM_001013191	Cbfβ	Core-binding factor, beta subunit			−3.92**
NM_053304	Col-1α1	Collagen, type I, alpha 1	3.19**		
NM_053356	Col-1α2	Collagen, type I, alpha 2	4.06**		
NM_032085	Col-3α1	Collagen, type III, alpha 1	5.00**	3.32*	
NM_001135009	Col-4α1	Collagen, type IV, alpha 1	2.04**	−4.45*	
NM_031622	Mapk6	Mitogen-activated protein kinase 6	2.21**		
NM_178866	Igf1	insulin-like growth factor 1, transcript variant 2	2.30*		
NM_001082479	Igf1	Insulin-like growth factor 1, transcript variant 4	2.24**		
NM_053329	Igfals	Insulin-like growth factor binding protein, acid labile subunit	2.02*		2.20*
NM_013144	Igfbp1	Insulin-like growth factor binding protein 1		−2.63*	
NM_001012118	Osr2	Odd-skipped related 2 (Drosophila)	2.75*	3.27**	
NM_001108550	Postn	Periostin, osteoblast specific factor	6.34**	3.07*	
NM_017044	Pth	Parathyroid hormone		2.46**	
NM_012636	Pthlh	Parathyroid hormone-like hormone	6.47**		
NM_020073	Pth1r	Parathyroid hormone 1 receptor	2.20**		

* = *P*<0.05,

** = *P*<0.01, - = down-regulated (E-Group versus S-Group samples).

**Table 3 pone-0032356-t003:** Genes with known or suspected roles in bone development or remodeling [3], [35], [36].

			Fold-change		
Genebank	Symbol	Description	1W	2W	4W
NM_198761	Adamts5	ADAM metallopeptidase with thrombospondin type 1 motif, 5	2.13**		
NM_001014008	Aspn	Asporin	6.18**	4.26**	
NM_031323	Bmp1	Bone morphogenetic protein 1	2.72*		
NM_017105	Bmp3	Bone morphogenetic protein 3	2.25*		
NM_012827	Bmp4	Bone m orphogenetic protein 4	2.24*		
NM_001108168	Bmp5	Bone morphogenetic protein 5	2.19**	2.43**	
NM_030849	Bmpr1a	Bone morphogenetic protein receptor, type IA		2.19**	
NM_013146	Cald1	Caldesmon 1	2.34**		
NM_012929	Col-2α1	Collagen, type II, alpha 1	2.35**		
NM_134452	Col-5α1	Collagen, type V, alpha 1	3.91**		
NM_053488	Col-5α2	Collagen, type V, alpha 2	4.17**		
NM_021760	Col-5α3	Collagen, type V, alpha 3	2.08*		
NM_001100741	Col-6α2	Collagen, type VI, alpha 2	2.21*		
NM_001107100	Col-8α1	Collagen, type VIII, alpha 1	2.35*		
NM_001100842	Col-9α1	Collagen, type IX, alpha 1	2.07*		
NM_001108675	Col-9α2	Collagen, type IX, alpha 2	2.90**		
NM_001108611	Co-l9α3	Procollagen, type IX, alpha 3	2.96**		
NM_212528	Col-11α2	Collagen, type XI, alpha 2	2.64**		
NM_001015033	Col-16α1	Collagen, type XVI, alpha 1	3.02**		
NM_053489	Col-18α1	Collagen, type XVIII, alpha 1	2.83**		
NM_198747	Col-27α1	Collagen, type XXVII, alpha 1	2.64**		
NM_053357	Ctnn-β1	Catenin (cadherin associated protein), beta 1	2.27*		
NM_031764	Ddr2	Discoidin domain receptor tyrosine kinase 2	2.05**		
NM_012712	Fgfr2	Fibroblast growth factor receptor 2, transcript variant a	−3.82*		
NM_053429	Fgfr3	Fibroblast growth factor receptor 3	2.49**		
NM_001109165	Fgfbp3	Fibroblast growth factor binding protein 3	2.11*	2.48**	3.04**
NM_138850	Fap	Fibroblast activation protein, alpha	2.16*		
NM_053428	Fgf13	Fibroblast growth factor 13			2.51*
NM_053708	Gbx2	Gastrulation brain homeobox 2	−3.60**	2.49**	
NM_024352	Mst1	Macrophage stimulating 1 (hepatocyte growth factor-like)			2.07**
NM_198768	Igsf10	Immunoglobulin superfamily, member 10	2.89**		
NM_053470	Runx2	Runt related transcription factor 2 (Runx2)			−2.35**
NM_130425	Runx3	Runt-related transcription factor 3 (Runx3)		−2.86**	
NM_013082	Sdc2	Syndecan 2	2.55**	2.18**	
NM_001100700	Sfrp2	Secreted frizzled-related protein 2	3.08**	2.89*	
NM_021578	Tgf-β1	Transforming growth factor, beta 1		−3.46*	
NM_031716	Wisp1	WNT1 inducible signaling pathway protein 1	2.54**		
NM_001170483	Wisp3	WNT1 inducible signaling pathway protein 3	−2.96**	−3.61**	

* = *P*<0.05,

** = *P*<0.01, - = down-regulated (E-Group versus S-Group samples).

Closer examination indicates that the mRNA expression levels of some pro-inflammatory cytokines and their receptors, including IL1f8, IL1rl1, Tnfsf9, Tnfsf11/RANKL, Tnfrsf11b/OPG, and Mmps [24]–[26], increased as early as 1 week post-surgery. Furthermore, mRNA expression of anti-inflammatory cytokines, such as IL10, Ifn-α4, Socs3, and Tgf-β [27]–[30], decreased in the early post-surgical stages, confirming that cytokines were involved in the inflammation response within the osteoarthritic subchondral bone.

Another interesting finding was that the vast majority of the DE genes coding for C-C, C-X-C, and C-X3-C chemokines (Ccl2/MCP1, Ccl3/MIP-1α, Ccl4/MIP-1β, Ccl7/MCP-3, Ccl-11, Ccl19, Cxcl9, Cxcl10, Cxcl11, Cxcl13, Cx3cl1, Cmklrl) and their receptor (Ccr5) were unexpectedly down-regulated at 1 week post-surgery. The exceptions to this trend were the up-regulation of mRNA levels of Ccl7 at 2 and 4 weeks post-surgery and of Ccr9 at 2 weeks post-surgery (**Table 4**). Similarly, a subset of the DE genes encoding pro-inflammatory cytokines (IL18, IL18 bp, Ifn-γ, Jak3, Sh2d2a, Stat1, Stat3, Ttrap, Vegfa) and their receptors (IL2rβ, IL7r, Tnfrsf9) were also down-regulated (**Table 5**) at 1 week post-surgery. These genes are thought to play important roles in OA pathophysiology and in subchondral cell migration [31]. Further studies are required to determine the pathophysiological roles of these chemokines and cytokines in osteoarthritic subchondral bone.

**Table 4 pone-0032356-t004:** DE genes encoding C-C, C-X-C, C-X3-C chemokines and their receptors [44]–[51].

			Fold-change		
Genebank	Symbol	Description	1W	2W	4W
AF079313	Ccl2 (MCP-1)	Monocyte chemoattractant protein-1 gene, 5′ flanking sequence	−5.71**	−3.07**	
NM_013025	Ccl3 (MIP-1α)	Chemokine (C-C motif) ligand 3	−2.28**		
NM_053858	Ccl4 (MIP-1β)	Chemokine (C-C motif) ligand 4	−4.08**		
NM_001007612	Ccl7 (MCP-3)	Chemokine (C-C motif) ligand 7		2.79*	2.38**
NM_019205	Ccl11	Chemokine (C-C motif) ligand 11	−2.05*		
NM_001108661	Ccl19	Chemokine (C-C motif) ligand 19	−4.52**		
NM_145672	Cxcl9	Chemokine (C-X-C motif) ligand 9	11.37**	-	
NM_139089	Cxcl10	Chemokine (C-X-C motif) ligand10	−12.08**		
NM_182952	Cxcl11	Chemokine (C-X-C motif) ligand11	−9.18**		
NM_001017496	Cxcl13	Chemokine (C-X-C motif) ligand13	−3.28*		
NM_134455	Cx3cl1	Chemokine (C-X3-C motif) ligand 1	−2.30**		
NM_053960	Ccr5	Chemokine (C-C motif) receptor 5	−2.00*		
NM_172329	Ccr9	Chemokine (C-C motif) receptor 9		2.19*	
NM_022218	Cmklr1	Chemokine-like receptor 1		−2.68**	

* = *P*<0.05,

** = *P*<0.01, - = down-regulated (E-Group versus S-Group samples).

**Table 5:** Inflammatory DE genes encoding cytokines and their receptors [24]–[26], [29], [31].

			Fold-change		
Genebank	Symbol	Description	1W	2W	4W
NM_001108570	IL1f8	Interleukin 1 family, member 8		2.51**	2.08*
NM_013037	IL1rl1	Interleukin 1 receptor-like 1, transcript variant 1,			2.03*
NM_013195	IL2rβ	Interleukin 2 receptor, beta	−2.67**		
NM_001106418	IL7r	Interleukin 7 receptor		−2.76*	
NM_012854	IL10	Interleukin 10	−2.00*		
NM_145789	IL13rα1	Interleukin 13 receptor, alpha 1		2.08*	
NM_019165	IL18	Interleukin 18	−2.74**		
NM_053374	IL18bp	Interleukin 18 binding protein	−2.35*		
NM_001106667	Ifn-α4	Interferon, alpha 4	−2.03*	−2.45*	
NM_138880	Ifn-γ	Interferon gamma	−2.73*		
NM_012855	Jak3	Janus kinase 3	−2.33*	−2.67*	
NM_032612	Stat1	Signal transducer and activator of transcription 1, transcript variant alpha	−2.38*		
NM_012747	Stat3	Signal transducer and activator of transcription 3			−4.42**
NM_053565	Socs3	Suppressor of cytokine signaling 3		−2.04*	
NM_001025773	Tnfrsf9	Tumor necrosis factor receptor superfamily, member 9	−2.66*		
NM_181384	Tnfsf9	Tumor necrosis factor (ligand) superfamily, member 9		2.17*	2.25*
NM_001034947	Ttrap	Traf and Tnf receptor associated protein			−2.25*
NM_031836	Vegfa	Vascular endothelial growth factor A, transcript variant 1	−2.71*		

* = *P*<0.05,

** = *P*<0.01, - = down-regulated (E-Group versus S-Group samples).

### Functional classification of the DE genes

**Figure 4:**
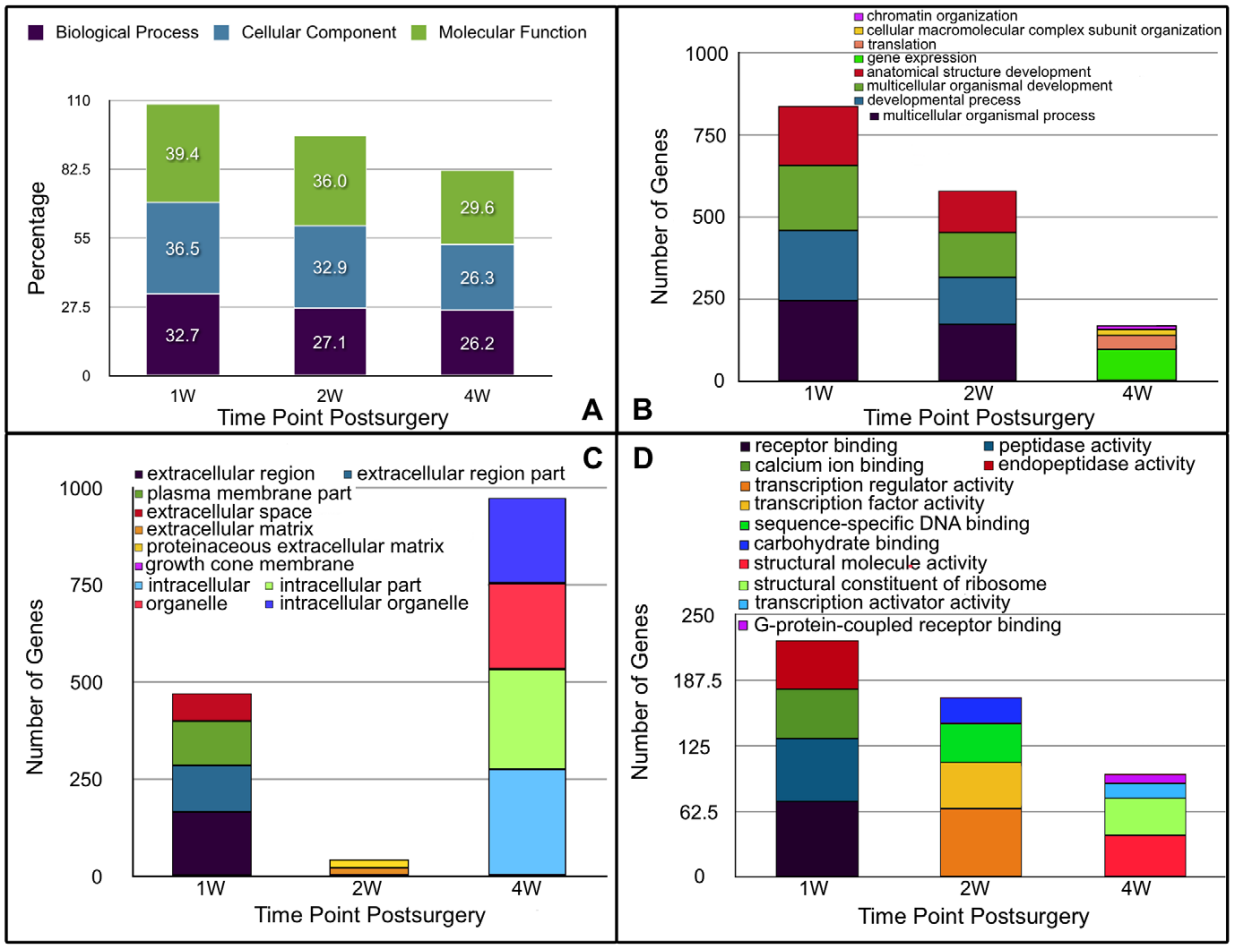
The distribution of the DE genes at each post-surgical time point is described based on the three gene ontology categories and the numbers of DE genes in the first four gene functional classifications of each gene ontology category. **A**. The distribution of DE genes was similar among the three gene ontology categories at each post-surgical time point. **B**. The functional classifications of the dysregulated genes involved in the biological processes category and the numbers of dysregulated genes in the first four functional classifications at each post-surgical time point. **C**. The functional classifications of the dysregulated genes involved in the cellular component category and the numbers of dysregulated genes in the first four functional classifications at each post-surgical time point. **D**. The functional classifications of the dysregulated genes involved in the molecular function category and the numbers of dysregulated genes in the first four functional classifications at each post-surgical time point.

Gene ontology (GO, http://www.geneontology.org) analyses were performed to obtain more detailed functional information regarding the DE genes. These analyses were based on *Rattus norvegicus* annotations for three gene ontology categories (biological process, cellular component, or molecular function). The distributions of the annotated DE genes involved in the three functional categorizations at each time point were similar to each other (**Figure 4A** **)**, suggesting that the dysregulation in gene expression involved in these categories occurred in parallel with disease progression in the subchondral bone of the osteoarthritic rat.

Of the 730 annotated genes that were significantly involved in a specific biological process at 1 week post-surgery, 33.7% were involved in multicellular organismal processes, 29.2% in developmental processes, 27.1% in multicellular organismal development, and 24.7% in anatomical structure development. These four categories were the most enriched (**Figure 4B**). Biological processes known to be related to OA pathways were studied in greater depth. The representative biological processes that were identified as significantly affected at 1 week post-surgery include the response to wounding, blood vessel development, skeletal system development, bone development, and activation of the plasma proteins involved in the acute inflammatory response (**Table 6**). Similarly, of the 527 annotated DE genes identified at 2 weeks post-surgery, 32.8% were involved in multicellular organismal processes, 27.5% in developmental process, 25.6% in multicellular organismal development, and 23.9% in anatomical structure development. The identified representative biological processes at 2 weeks post-surgery included the response to steroid hormone stimulus, skeletal system development, blood vessel development, and wound healing (**Table 7**). Of the 397 annotated DE genes at 4 weeks post-surgery, 24.7% were involved in gene expression, 10.3% in translation, 4.5% in cellular macromolecular complex subunit organization, and 3.3% in chromatin organization. Only three representative biological processes were identified at this time point, including bone development, positive regulation of hormone secretion, and calcitonin catabolic processes (**Table 8**). Detail information of the four most enriched categories can be found online in **Supplementary Table S3**.

**Table 6:** Representative biological processes enriched with DE genes identified at 1 week post-surgery.

GOID	GO term*	Number of genes	Fisher-Pvalue
GO:0032501	**multicellular organismal process**	246	0.0057
GO:0032502	**developmental process**	213	0.00009
GO:0007275	**Multicellular organismal development**	198	0.00003
GO:0048856	**anatomical structure development**	180	0.00012
GO:0009605	response to external stimulus	64	0.00284
GO:0009611	response to wounding	56	0.00141
GO:0001568	blood vessel development	41	0
GO:0048583	regulation of response to stimulus	40	0.03473
GO:0001501	skeletal system development	38	0.00001
GO:0048545	response to steroid hormone stimulus	38	0.02035
GO:0001944	vasculature development	42	0
GO:0048514	blood vessel morphogenesis	33	0.00001
GO:0042060	wound healing	30	0.00015
GO:0060348	bone development	27	0.00004
GO:0048584	positive regulation of response to stimulus	25	0.01695
GO:0001503	ossification	23	0.00071
GO:0030198	extracellular matrix organization	23	0
GO:0006816	calcium ion transport	19	0.03767
GO:0032101	regulation of response to external stimulus	19	0.04736
GO:0034097	response to cytokine stimulus	17	0.02595
GO:0006935	chemotaxis	16	0.00095
GO:0016055	Wnt receptor signaling pathway	14	0.03239
GO:0048705	skeletal system morphogenesis	14	0.00029
GO:0051216	cartilage development	14	0.0005
GO:0009612	response to mechanical stimulus	12	0.02122
GO:0032103	positive regulation of response to external stimulus	12	0.00556
GO:0051924	regulation of calcium ion transport	11	0.01044
GO:0001649	osteoblast differentiation	10	0.03949
GO:0030199	collagen fibril organization	9	0
GO:0060349	bone morphogenesis	9	0.00001
GO:0032963	collagen metabolic process	8	0.00368
GO:0002062	chondrocyte differentiation	7	0.00254
GO:0002541	activation of plasma proteins involved in acute inflammatory response	7	0.00649
GO:0051928	positive regulation of calcium ion transport	6	0.04434
GO:0060350	endochondral bone morphogenesis	6	0.0001
GO:0001569	patterning of blood vessels	5	0.00279
GO:0045669	positive regulation of osteoblast differentiation	5	0.03069
GO:0061035	regulation of cartilage development	5	0.00067
GO:0030574	collagen catabolic process	4	0.01807
GO:0032964	collagen biosynthetic process	4	0.03775
GO:0050918	positive chemotaxis	4	0.03775
GO:0050926	regulation of positive chemotaxis	4	0.02368
GO:0050927	positive regulation of positive chemotaxis	4	0.02368
GO:0060351	cartilage development involved in endochondral bone morphogenesis	4	0.00022
GO:0071345	cellular response to cytokine stimulus	4	0.03775
GO:0001958	endochondral ossification	3	0.01527

*GO term in bold are the four most enrich biological processes.

**Table 7:** Representative biological processes enriched with DE genes identified at 2 weeks post-surgery.

GOID	GO term*	Number of genes	Fisher-Pvalue
GO:0032501	**multicellular organismal process**	173	0.04764
GO:0032502	**developmental process**	145	0.01263
GO:0007275	**multicellular organismal development**	135	0.00601
GO:0048856	**anatomical structure development**	126	0.00412
GO:0048545	response to steroid hormone stimulus	28	0.03465
GO:0001501	skeletal system development	23	0.00673
GO:0001568	blood vessel development	20	0.02913
GO:0001944	vasculature development	20	0.03454
GO:0042060	wound healing	18	0.02142
GO:0043627	response to estrogen stimulus	17	0.01539
GO:0006816	calcium ion transport	15	0.03209
GO:0034097	response to cytokine stimulus	15	0.0068
GO:0060348	bone development	15	0.02454
GO:0030198	extracellular matrix organization	12	0.00007
GO:0032355	response to estradiol stimulus	12	0.01553
GO:0051216	cartilage development	11	0.00111
GO:0048705	skeletal system morphogenesis	10	0.00265
GO:0051924	regulation of calcium ion transport	9	0.00964
GO:0043406	positive regulation of MAP kinase activity	8	0.04488
GO:0048706	embryonic skeletal system development	7	0.00989
GO:0000187	activation of MAPK activity	6	0.045
GO:0048704	embryonic skeletal system morphogenesis	6	0.01051
GO:0060349	bone morphogenesis	5	0.00428
GO:0051926	negative regulation of calcium ion transport	4	0.00775
GO:0030199	collagen fibril organization	3	0.03856
GO:0001569	patterning of blood vessels	3	0.03856
GO:0030513	positive regulation of BMP signaling pathway	2	0.04539
GO:0033687	osteoblast proliferation	2	0.03149
GO:0051280	negative regulation of release of sequestered calcium ion into cytosol	2	0.00355
GO:0010523	negative regulation of calcium ion transport into cytosol	2	0.01024
GO:0090090	negative regulation of Wnt receptor signaling pathway through beta-catenin	2	0.03149

*GO terms in bold are the four most enriched biological processes.

**Table 8:** Representative biological processes enriched with DE genes identified at 4 weeks post-surgery.

GOID	GO term*	Number of genes	Fisher-Pvalue
GO:0010467	**gene expression**	98	0.02722
GO:0006412	**translation**	41	0
GO:0034621	**cellular macromolecular complex subunit organization**	18	0.01368
GO:0006325	**chromatin organization**	13	0.04592
GO:0060348	bone development	12	0.02852
GO:0046887	positive regulation of hormone secretion	6	0.02371
GO:0010816	calcitonin catabolic process	1	0.04494

*GO terms in bold are the four most enriched biological processes.

Further analysis revealed many common representative biological processes at 1 and 2 weeks post-surgery, however, annotated genes involved in the common biological processes were quite different between the this two time points. The only common biological process to all three time points was bone development. This finding suggests that similar biological processes involved in subchondral bone alterations occurred at 1 and 2 weeks post-surgery. Additionally, these findings demonstrate that subchondral bone remodeling occurred throughout OA pathogenesis in this model.

Interestingly, of the annotated DE genes involved in the cellular component category, the greatest number was predominantly involved in the extracellular component category at both 1 and 2 weeks post-surgery. At 4 weeks post-surgery, however, the greatest number of DE genes was involved in the intracellular component category (**Figure 4C**), highlighting the importance of extracellular processes in the very early stages of OA. The classifications of the molecular functions that were enriched in the set of DE genes were quite different among the three time points (**Figure 4D**), suggesting that time-dependent molecular changes occurred in osteoarthritic subchondral bone.

### DE genes validated by real-time PCR

**Figure 5:**
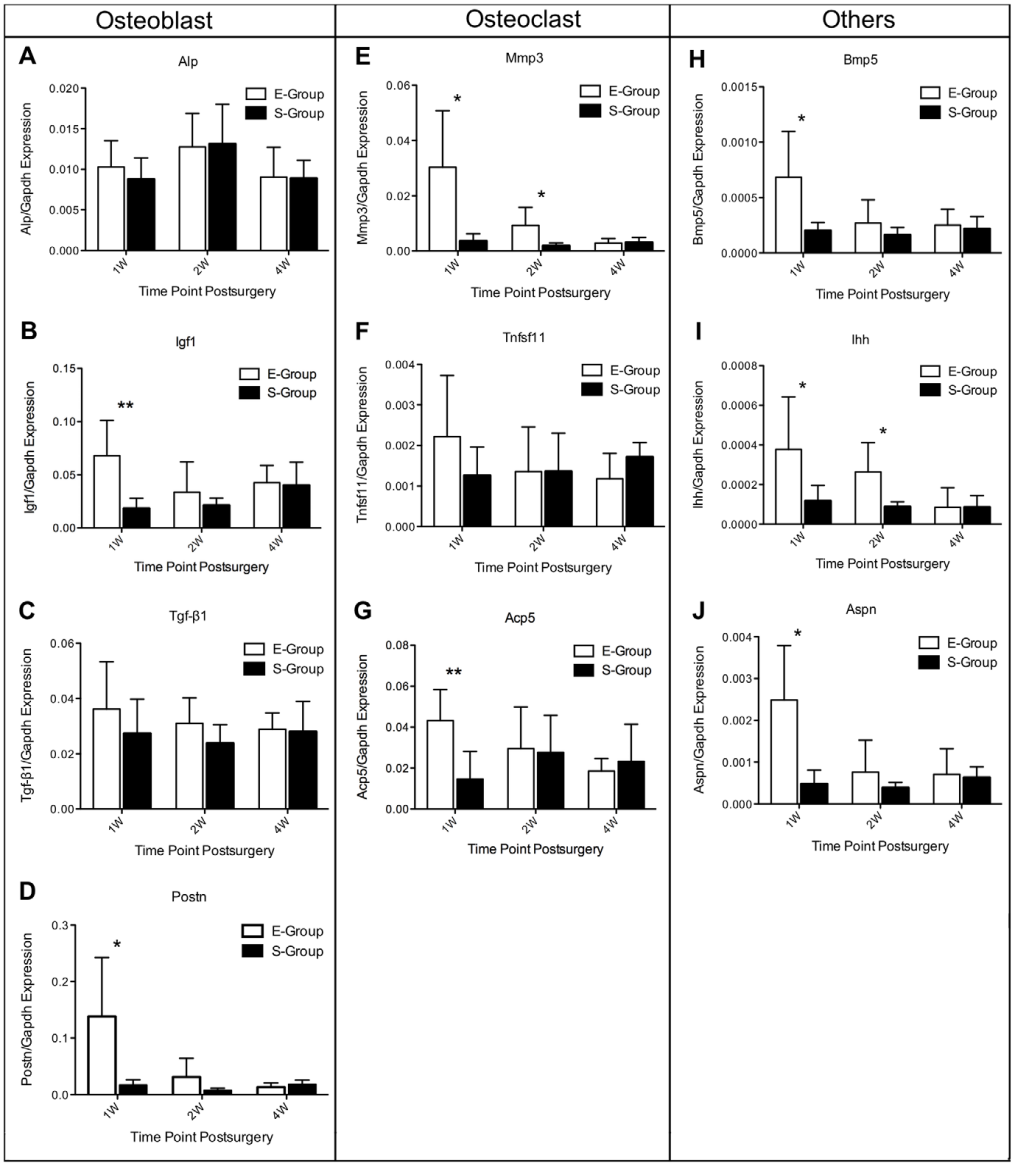
Gene expression patterns of Alp, Igf1, Tgf-β1 and Postn (genes involved in osteoblasts differentiation and function), Mmp3, Tnfsf11 and Acp5 (genes involved in osteoclasts differentiation and function), and Bmp5, Aspn, Ihh (known OA-related genes), evaluated by real-time polymerase chain reaction (PCR). The expression profiles of each gene matched the tested probes at a minimum of two time points. A similar degree of variability was observed at other time points in the real-time PCR results without statistically significant differences between groups. The values are the mean and SEM of the gene expression levels in 5 animals (separate from the animals used for microarray analyses), as determined by ΔCt analysis, normalized to GAPDH expression, and relative to the expression levels of sham-operated controls. * *p*<0.05, ** *p*<0.01 (versus sham-operated controls).

The DE genes indicated by our microarray analyses to be involved in osteoblast differentiation and function (e.g., Alp, Igf1, Tgf β1 and Postn), those involved in osteoclast differentiation and function (e.g., Mmp3, Tnfsf11 and Acp5), and known OA genes (e.g., Bmp5, Aspn and Ihh) were confirmed by real-time PCR. As shown in **Figure 5**, gene expression patterns for Mmp3, Acp5, and Igf1 precisely matched the expression patterns obtained from microarray analysis, and these three genes are involved in the differentiation and function of osteoclasts or osteoblasts. However, Alp, Tgf-β1 and Tnfsf11 were not differentially expressed as determined by real-time PCR at any time point. Of the remaining genes, real-time PCR results matched the microarray analysis for at least one time point. The expression profiles of each gene that was confirmed by real-time PCR matched the results obtained from the microarray analysis for at least at two time points, with the exceptions of Alp and Ihh. These differences were perhaps due to the heterogeneity of osteoarthritis between individual animals and potential false positives in the microarray data. However, the expression profiles of Mmp3 and Acp5 were validated by real-time PCR, the results of which were identical to the profiles that were determined using microarray analysis. Although most target genes only matched the tested probes at two time points, it is important to note that a similar degree of variability was observed at other time points in the real-time PCR results without statistically significant differences between groups. Overall, these results indicated that our microarray data accurately reflect gene expression patterns. Furthermore, these data indicate that subchondral bone remodeling occurs in a time-dependent manner and in parallel with disease progression.

### Validation of gene expression at the protein level

**Figure 6:**
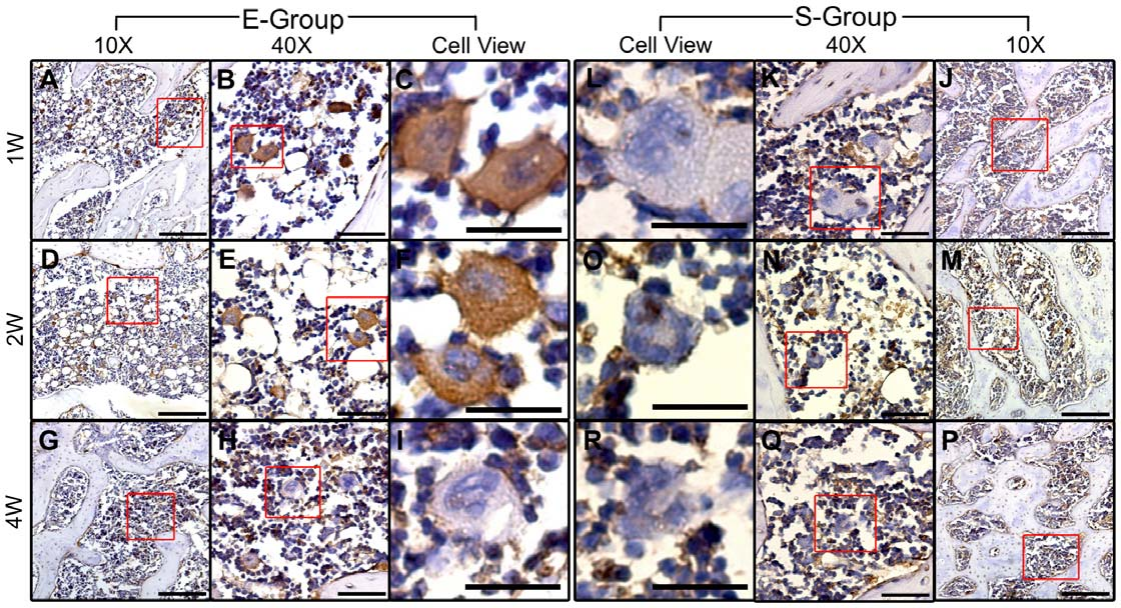
Evaluation of the expression levels of Mmp3 in the subchondral bone of the E-Group and the S-Group using immunohistochemistry staining. The antibody against Mmp3 was used to assess the spatial and temporal expression of the protein by colorimetric detection (brown precipitate). The mononuclear cells of both groups appeared positive for the same levels of Mmp3 at 1, 2 and 4 weeks post-surgery. The polynuclear giant cells in the subchondral bone of the E-Group expressed Mmp3 in their cytoplasm 1 and 2 weeks post-surgery, but not at 4 weeks, wheras those of the S-Group appeared negative at all three time points post-surgery. Specifically, obvious differences were discovered in the E-Group between 1 week and 2 weeks post-surgery, including stronger positive signals and more positive cells at 1 week compared with 2 weeks post-surgery. All sections were counterstained with hematoxylin (blue stain). The scale bars for 10X, 40X and Cell-view are 200 µm, 50 µm, and 50 µm respectively.

To gain further insight and independent support for the results of our microarray analysis, immunohistochemistry staining was performed to examine the protein levels of Mmp3 and Aspn in tissue sections from the E-Group and the S-Group. Mmp3 was chosen for investigation because it is an important factor in bone metabolism [32]–[36]. Mmp3 protein expression levels were markedly increased in the E-Group compared with the S-Group at 1 and 2 weeks post-surgery, whereas no significant difference was observed between these two groups 4 weeks post-surgery. These results are in complete agreement with the expression levels of this gene determined by real-time PCR. Specifically, a clear difference in Mmp3 expression was identified in the E-Group between 1 and 2 weeks post-surgery, including a stronger positive signal and more positive cells that were positive for Mmp3 at 1 week post-surgery. A subset of polynuclear giant cells and a subset of smaller mononuclear cells in the subchondral bone area of E-Group samples were strongly positive for cytoplasmic Mmp3 at 1 and 2 weeks post-surgery. In the same anatomical region in the S-Group samples, these cells appeared negative for Mmp3. As indicated by these histological analyses, no obvious cartilage damage was observed in the E-Group at 1 week post-surgery. The results from immunohistochemistry and real-time PCR analyses, however, strongly support the hypothesis that remarkable changes occur in the subchondral bone and precede significant articular cartilage degeneration (Figure 6). Nevertheless, differential expression of Aspn protein between the E-Group and the S-Group was not observed in the subchondral bone at any time point (results not shown). Aspn mRNA levels, however, were observed to be significantly increased at 1 week and 2 weeks post-surgery based on microarray analyses and real-time PCR. However, gene expression levels do not necessarily predict protein levels due to alternative transcriptional and translational steps and protein degradation. Negative control cells stained with secondary antibody alone confirmed the specificity of the primary antibody (results not shown).

## Discussion

OA is a disease of the cartilage and bone, and studying the molecular changes of subchondral bone contributes to the general understanding the pathogenesis of OA [3]–[5]. Because there are no adequate available human samples that are available to be used in the study of OA in subchondral bone, an experimental rat OA model was used in this study. In this model, knee joint instability was surgically induced and shown to be 1) consistent with the post-traumatic OA in humans and 2) useful for genetic analyses [20]–[22]. One major difficulty for gene expression research in the subchondral bone is RNA degradation during the isolation procedure. High-quality RNA from subchondral bone samples is the foundation of reliable gene expression analyses, such as microarray and quantitative RT-PCR analyses. Here, we describe for the first time a simple and effective method for RNA isolation from this tissue. Using a micro-electric burnishing instrument, the femur condyle was separated in liquid nitrogen to obtain the subchondral bone sample, effectively avoiding RNA degradation. There was, however, one primary limitation of this method: the removal of articular cartilage and epiphyseal plate tissue was primarily judged subjectively by the operator who viewed the tissues under a microscope during the operation. Considering the organized structure of the rat knee [7], [21], the identification of the different tissue compartments of this joint is straightforward, this limitation can be considered negligible.

Bone remodeling is regulated by the balanced processes of osteoclast-mediated bone resorption and osteoblast-mediated bone formation. Disequilibrium of this balance leads to dysregulated bone tissue remodeling, and can result in excessive bone loss or extra bone formation and consequent skeletal disease [37], [38]. Previous studies have demonstrated that early subchondral bone loss is followed by increased bone density and OA progression [5], [21], [39]. In our study, as shown in **Tables 1** **–** [Table pone-0032356-t002] [Table pone-0032356-t003] [Table pone-0032356-t004] **5**, we identified a group of DE genes that play roles in osteoclast and osteoblast differentiation and function in the early stages of OA in this model. These data provide additional evidence supporting the conclusion that an altered phenotype of subchondral osteoblasts and osteoclasts contribute to OA [11], [15], [16], [19], [33]–[35], [38].

Bone tissue remodeling involves the synthesis and degradation of the collagenous framework of bone. Fibrous type I collagen is the major structural component of bone, and alterations in bone collagen metabolism are detrimental to the structural properties of bone [3]. Abnormalities of the type I collagen homotrimer have been observed in osteoblasts cultured from the bones of patients with advanced OA. This finding was primarily attributable to increased Col-1α1 expression and unchanged Col-1α2 expression. This abnormal Col1α1: Col1α2 ratio generated a matrix that blunted mineralization in OA osteoblasts [3], [40], [41]. Consistent with these results, we observed increased expression of both Col-1α1 and Col-1α2 at 1 week post-surgery in our study. However, changes in the expression levels of Col-1α2 were slightly greater than for Col-1α1, which exhibited different features of bone type I collagen expression during the first stage of the rat OA model.

In addition, our study revealed a group of up-regulated collagen mRNAs at 1 week post-surgery. However, José Ramón et al. recently reported that several collagens (i.e., Col4a1, Col4a2, Col8a1, Col10a1, and Col11a1) were clearly down-regulated in bone marrow mesenchymal stem cells from patients with advanced OA [42]. Whether these discrepant results are due to the species used or the disease stage examined is unclear. In particular, and in partial agreement with the results of this previous study, Col4a1 expression increased at 1 week post-surgery and then decreased at 2 weeks post-surgery in our study. These results indicate that collagen-related metabolic changes occurred in a time-dependent manner in this OA model.

Collagen II is a major and uniformly distributed component of cartilage, and its destruction leads to cartilage degradation. Up-regulated collagen II expression was observed at 1 week post-surgery in our study. It has been suggested that collagen II is expressed and produced by mesenchymal cells in the subchondral bone and is then transported to the overlying cartilage [43]. This transport would occur via clefts or channels in the subchondral bone for the purpose of protecting the cartilage from erosion during the very early stages of OA [4], [8], [9], [11]. Klatt AR et al. reported that collagen II may be involved in cartilage degradation by inducing MMPs and pro-inflammatory cytokines [44]. Given that MMPs and pro-inflammatory cytokines were also up-regulated in our model, we conclude that collagen II expressed in the subchondral bone cells may also be involved in subchondral bone remodeling by mediating and modulating extracellular matrix (ECM) degradation. Further studies will be required to fully understand the pathophysiological roles of MMPs and pro-inflammatory cytokines in osteoarthritic subchondral bone.

Chemokines are known to play important roles in chronic inflammatory diseases, such as rheumatoid arthritis (RA) and OA. Chemokines are divided into four subgroups: C, CC, CXC, and CX3C, according to the position of their cysteine residues [45]. Chemokines are abundant within inflamed tissues in joints [46] and affect the activation and migration of circulating cells into tissue [47]. Furthermore, chemokines produced in subchondral bone can directly or indirectly induce cartilage deterioration and bone remodeling during OA progression [48], [49]. Recent studies have reported that the expression of chemokines and their receptors is increased in cartilage, bone, synovial membrane and synovial fluid samples from osteoarthritic patients [48]–[53]. However, the majority of these results was obtained from patients with advanced OA and does not reveal the molecular changes associated with the early stages of OA. The present study, however, demonstrates for the first time that the vast majority of chemokines and their receptors exhibit significantly down-regulated mRNA expression in the early stages of the rat model of OA. Similarly, a subset of pro-inflammatory cytokines and their corresponding receptors that are involved in chemokine signaling pathways, including IL18, IL18bp, IL2rβ, IL7r, Ifn-γ, Tnfrsf9, Ttrap, Jak3, Stat1, and Stat3 [30], also exhibited reduced expression at 1 week post-surgery. Cytokines and growth factors are known to actively participate in functional alterations of the synovium, cartilage and subchondral bone. Furthermore, these molecules are produced by cells in osteoarthritic joint tissues [31], and the relationship between inflammatory cytokines and chemokines is complex. Guo H et al. demonstrated the production and expression of chemokines and their receptors in human chondrocytes in response to IL-1 and TNFα treatment [51]. Down-regulation of the expression of these cytokines may negatively affect chemokine signaling and may influence the Jak-Stat signaling pathway. It is by these mechanisms that cytokines may modulate different cell functions, such as cytokine production, migration, cellular proliferation and apoptosis [30], [54], [55].

These gene expression data do not directly prove that inflammation was reduced in the subchondral bone in the very early stages of OA; however, several lines of evidence support this conclusion: 1) a reduction in inflammation may be a protective mechanism in response to external mechanical stimuli on the subchondral bone; 2) subchondral bone is in a different inflammatory joint compartment during the very early stages of OA, one that likely expresses fewer inflammatory molecules, such as chemokines and pro-inflammatory cytokines; 3) given that they lack chemokine and chemokine receptor expression, mesenchymal cells may have a more limited potential to diffuse into the overlying cartilage through the subchondral cracks [46]–[48]; 4) chemokines and their receptors are not uniquely restricted to inflamed subchondral bone but can alter bone metabolism [54]; 5)the expression patterns of chemokines and their receptors were observed to be distinct between the early and late stages of OA when the results of previous studies of late-stage OA were considered [48]–[52]. Elucidating the function of chemokines and their receptors may provide novel and important insights into the pathophysiology of subchondral bone in OA.

Although our findings conflict with the results of certain previous studies [48]–[52], other studies have also reported that the expression of chemokines and their receptors is reduced in OA. Haringman et al. demonstrated that CCR1 and CCR5 expression in peripheral blood cells from arthritis patients was significantly decreased when compared with healthy controls [55]. Furthermore, a group of chemokines, including Ccl2, Ccl22, Ccl24, Ccl27, Cxcl7, Cxcl10, Cxcl12 and Xcl1, exhibited significantly reduced expression levels in samples from OA patients compared with samples of normal synovial fluid [49]. Both of these findings are in partial agreement with our results. Interestingly, we observed that the expression of Ccl2, Ccl7, Ccr9 and certain pro-inflammatory cytokines (i.e., IL1f8, IL1rl1, IL13rα1, and Tnfsf9) began increasing at 2 or 4 weeks post-surgery. At the same time, the expression of certain anti-inflammatory cytokines, such as IL10, Ifn-α4, Tgf-β, and Socs3 [27]–[30], decreased. In particular, Ccl2 expression was reduced at 1 week post-surgery and was increased at 2 weeks post-surgery, indicating that changes in chemokine expression were time-dependent. Furthermore, these results suggest that inflammation increases in the later stages of OA. To summarize, our study strongly supports the idea that inflammation actively participates in subchondral bone remodeling in a rat model of OA.

There is clear evidence that chemokines stimulate various cell types in the osteoarthritic joint, thereby promoting these cells to produce cytokines and metalloproteinases (Mmps) [48]–[53]. Although we observed a reduced expression of chemokines in our study, Mmp expression (i.e., Mmp1b, 2, 3, 12, 13, 14, 16, 23, and 24) was increased as early as 1 week post-surgery, further supporting the conclusion that Mmp expression increases bone resorption in OA [32]–[36]. These results were not unexpected given the complex regulatory and signaling networks involved in the regulation of gene expression.

Mmp3 is a stromelysin and has broad activity on a variety of extracellular matrix molecules, including proteoglycans, fibronectin, gelatin, collagen types IV and IX, and laminin [36]. Many studies have reported that Mmp3 is expressed in various bone cell types and actively participates in bone resorption and formation [8], [32]–[36], [56]. Kusano et al. reported that Mmp2, Mmp-3, and Mmp-13 are expressed in mouse osteoblastic cells and that their expression is markedly enhanced by treatment with IL-1α and IL-6 treatment [32]. Meikle et al. demonstrated that human osteoblasts synthesize Mmp1, Mmp-3 and Mmp-9 in vitro [56]. Moreover, Mmp3 in human osteoblasts and osteoclasts actively participates in bone formation [22]. It has also been shown that Mmp activity is indispensable for the migration of (pre)osteoclasts to future resorption sites [57]. Following IL-1α stimulation, rabbit osteoclasts expressed Mmp1, Mmp-2, Mmp-3, and Mmp-9 and TIMP-1. The Mmps were then released into the sub-osteoclastic resorption zone, where they participated in bone collagen degradation [58]. In particular, our study demonstrated the up-regulation of Mmp3 expression in polynuclear cells and mononuclear cells at both the mRNA and protein levels at 1 and 2 weeks post-surgery. Although it is unclear what these cells are in the subchondral bone, polynuclear giant cells and mononuclear cells that express Mmp3 in the subchondral bone certainly play critical roles in the early pathophysiology of OA. Further study is required to determine the exact functions of these cells in OA. To our knowledge, these results represent the first time that the expression levels of groups of chemokines and Mmps were shown to be down-regulated and up-regulated, respectively, in subchondral bone during the early stages of an animal model of OA. These results could help us better understand the events that occur in subchondral bone during the early stages of OA and may be helpful in the identification of molecular diagnostic markers and therapeutic targets.

OA has been correlated with alterations in joint biomechanics [59]. Therefore, molecular changes in response to mechanical stimuli are likely etiologic for OA at early stages, which may lead to subsequent pathologic changes in the cartilage and subchondral bone. Indian Hedgehog (Ihh) is considered a critical mediator in transducing mechanical signals to stimulate chondrocyte proliferation [60], [61]. Repeated mechanical loading triggers the expression of Ihh, which in turn increases the number of replicating mesenchymal cells and the amount of the cartilage that is formed [62]. Moreover, Hh signaling is activated in OA and higher levels of Hh signaling in chondrocytes induce a more severe osteoarthritis phenotype. Furthermore, blocking Hh can be used as a therapeutic approach to inhibit articular cartilage degeneration in rat models of OA [63].

We have demonstrated that Ihh expression increased at 1 and 2 weeks post-surgery and was unchanged at 4 weeks post-surgery. Our study provides evidence that Ihh is an important mediator in the transduction of mechanical signals, causing chondrocyte proliferation and hypotrophy. This effect was a result of the surgically induced force imbalance within the joint. Our study is the first to show increased Ihh expression in subchondral bone in a rat model of OA as early as 1 week post-surgery. At this stage, morphologic analyses revealed almost no alteration in the articular cartilage. Based on an unusual expression profile of Ihh, it is possible that Ihh immediately responded to the abnormal forces on the joint, causing serial molecular changes in the subchondral bone. The Ihh signal may then have been transduced, triggering proliferation and hypotrophy signals within the chondrocytes, and, finally, degeneration of the articular cartilage, which is known to occur in late OA in both animals and humans. The early stimulation of Ihh in subchondral bone may be a driving mechanism of osteoarthritis and may, therefore, constitute a perfect therapeutic target for blocking the progress of osteoarthritis at a very early stage, when the cartilage is still intact.

Furthermore, it has been reported that Hh indirectly regulates Adamts5 via Hh-mediated expression of RUNX2, which is thought to be involved in hypertrophic changes in chondrocytes and the destruction of the cartilage matrix [63]. Recent research has demonstrated that the joints of ADAMTS5−/− mice that received transection of the medial meniscotibial ligament (to induce OA) were protected from cartilage damage. The joints of these mice also exhibited only minor changes to the subchondral bone structure when compared with the substantial changes observed in wild type mice that were subject to the same surgery [64] The microarray data in our study, however, indicate that Adamts5 and Runx2 were down-regulated at 1 and 4 weeks post-surgery, respectively. This finding suggests that Ihh may function via a pathway other than Adamts5 and Runx2, one that leads to chondrocyte hypotrophy and cartilage loss. The mechanism by which mechanical loading stimulates Ihh signaling and initiates OA remains unknown and requires further research.

Periostin (Postn) is a secreted protein that was originally identified in mouse osteoblasts. It is reported to promote cell adhesion and migration, which is clearly detectable in the perichondrium surrounding developing bones. This fact suggests that Postn may play a role in the recruitment of cells to the chondrocyte lineage [65]–[67]. A recent study documented that Postn can respond to mechanical stimuli in vitro; this response was demonstrated when the Postn expression in an odontoblast cell line subjected to a mechanical force was significantly inhibited [68]. In addition, Postn is known to play an essential role in the progression of cardiac valve complex degeneration by inducing angiogenesis and the production of Mmps, including Mmp2 and Mmp13 [69]. Both of these Mmps were observed to be increased at 1 week post-surgery based on our microarray data. We also confirmed that the expression of Postn significantly increased 1 week after the surgery using real-time PCR. To the best of our knowledge, no previous research has focused on the correlation between OA and Postn. Based on the above data, we conclude that Postn expression may be immediately stimulated by the abnormal mechanical force due to the surgery, causing angiogenesis and metalloproteinase-induced cellular matrix degeneration, thus initiating cartilage loss. This finding might conflict with the previous research [68]; however, this difference may have occurred because in vitro and in vivo experiments occasionally give opposing results. More research needs to focus on the mechanism by which Postn responds to mechanical forces, how it initiates these reactions, and how it relates to OA.

Bone morphogenetic proteins (Bmps) have been shown to participate in many signaling processes, such as organogenesis, tissue differentiation and dorsal-ventral patterning. In humans, Bmps are known to act as fundamental regulators of bone and cartilage development and homeostasis and play a pivotal role in bone and cartilage growth and repair [70]–[74]. BMPs implanted into soft tissue in vivo cause the formation of heterotopic bone via an endochondral process [75]–[77]. Our microarray data indicate a dramatic increase in Bmp expression (including Bmp1, Bmp3, Bmp4, and Bmp5) in the subchondral bone in a rat model of OA as early as 1 week post-surgery. These significant changes suggest that the formation of osteophytes in OA joints may result from Bmp signaling.

Furthermore, Bmp5 plays a functional role in skeletal development and is expressed postnatally in the tibial and costochondral growth plate of rats. In these tissues, Bmp5 expression results in increased proliferation and cartilage matrix synthesis in chondrocytes [78], [79]. Studies of mice with Bmp5 cleavage mutation also indicate that Bmp signaling is an important part of mechanotransduction in the bone [78]. Consistent with these results, our data provide evidence that Bmp5 plays a critical role in mechanotransduction in bone. The expression of Bmp5 is increased at 1 week post-surgery, reflecting another mechanism by which mechanical force changes have extremely important effects and result in an immediate and widespread molecular response.

In summary, the results of previous studies were based on samples obtained from patients with late-stage OA or in vitro experiments. Neither of these models can precisely describe the molecular changes that occur in the early stages of OA in vivo. Our results were obtained by examining the early stages of an animal model of OA and were confirmed using both real-time PCR and immunohistochemistry. In addition to providing a simple and effective method to separate of subchondral bone sample from the knee joint in a rat model of OA, we report the gene expression profiles of subchondral bone in the rat OA model at multiple post-surgical time points. At the same time, our findings provide data on the time-course of the cell-level molecular changes in osteoarthritic subchondral bone. These findings strongly support an important role for subchondral bone remodeling in OA pathogenesis. Most importantly, we identified DE genes with known or suspected roles in bone development and remodeling. These identified genes may be used as novel diagnostic markers or as therapeutic targets for OA.

## Materials and Methods

### Rat model of osteoarthritis

Ninety 10-week-old male Sprague-Dawley rats (Laboratory Animal Center of Sun Yat-Sen University, China) weighing 300–325 g were used in the study (Animal quality certificate number: 0061858, 0062374, 0071053). The animals were divided equally into two groups: the experimental group (E-Group) and the sham-operated group (S-Group). Rats in the E-Group underwent open surgery. This surgery involved both a medial meniscectomy and a medial collateral ligament (MCL) transection with micro-scissors as described previously [21], [22]. All rats were anesthetized with isoflurane. After the surgical sites were shaved and sterilized, the right knee joints were operated upon using a medial parapatellar approach. The articular capsule was then washed with a sterile saline solution, and the incision was closed in two layers. The articular capsule was sutured intermittently and independently with 5-0 absorbable sutures (Jinhuan Medical Supplies Company, Shanghai, China). The skin was closed with interrupted sutures using 5-0 silk threads (Jinhuan Medical Supplies Company, Shanghai, China). The S-Group rats were subjected to a sham operation in which a similar incision was made, but no changes were made to the medial meniscus or the medial collateral ligament. Following the surgery, all animals were immediately treated with penicillin (North China Pharmaceutical Co., LTD, Shijiazhuang, Hebei Province, China) and tramadol (North China Pharmaceutical Co., LTD, Shijiazhuang, Hebei Province, China).

Every procedure was reviewed and approved by the Institutional Animal Care and Use Committee at the Sun Yat-sen University (Approval ID: 0048139).

### Specimen processing

The animals were sacrificed at 1, 2, and 4 weeks post-surgery. Fifteen animals were used for each time point in each treatment group. Five animals were used for histological analysis and immunohistochemistry; five were used for microarray analysis and another five were used for real-time PCR. Following disarticulation of the right knee joints of the 5 animals, the femurs were cleaned and washed with physiologic saline. The gross appearance of the distal femur was recorded using a digital camera (IXUS 960IS, Canon, Japan), and the distal section of each femur was fixed in 4% paraformaldehyde (Boster, China) in phosphate-buffered saline (PBS) for 24 h. The samples were then decalcified with 20% EDTA (pH 7.4) for 3 weeks at 4°C; the medium was changed every 3 days.

Following exarticulation of the right knee joints of the other ten animals, the femurs were rapidly fractured from the epiphyseal plate. The femoral condyles were then frozen in liquid nitrogen for subchondral bone separation and total RNA extraction.

### Histology

Following decalcification, the medial condyles of the femurs were embedded in paraffin wax and sectioned in the sagittal plane. Paraffin sections (5 µm thick) were stained with H&E, 0.5% safranin-O (Sigma Corp., St. Louis, MO) for glycosaminoglycans, 0.1% rapid green (Sigma Corp., St. Louis, MO) for collagen, and counterstained with Mayer's hematoxylin (Sigma Corp., St. Louis, MO) for nuclei. The images were captured with a Leica DMI 3000 B microscope (Germany) and software (Leica Application Suite V3).

### Subchondral bone separation and total RNA extraction

The femur condyles frozen in liquid nitrogen were placed in insulated boxes, which were also filled with liquid nitrogen to avoid RNA degradation. The condyles were then stabilized with a holding clamp. The articular cartilage and epiphyseal plate tissue of the femur condyle sample were cleared away with a micro-electric burnishing instrument (Strong90, New Power, Korea) and a drill (SDE-H37L1, Marathon; maximum RPM: 35,000) to isolate the bone (3,500 RPM). During this process, the samples were protected by liquid nitrogen, and an operative microscope (AXS, Shanghai Anxi Optical Equipment Manufacture Co., China) was used to ensure that the articular cartilage and epiphyseal plate tissues were removed.

Total RNA from the subchondral bone was extracted using an E.Z.N.A Total RNA Kit II (Omega Bio-Tek, Inc., USA) according to the manufacturer's instructions, including a DNase digestion step. The quantity and quality of RNA were assessed using a NanoDrop ND-1000 spectrophotometer (NanoDrop Technology, Rockland, DE) and agarose gel electrophoresis.

### Agilent microarray study

The RNA was further purified using RNeasy spin columns (Qiagen p/n 74104) according to the manufacturer's instructions. One microgram of total RNA was isolated from the subchondral bone of 5 E-Group and 5 S-Group knee joints for each time point. The RNA was amplified and labeled using the Agilent Quick Amp One-Color labeling kit (Agilent p/n 5190-0442). The RNA was then hybridized to Agilent Whole Rat Genome Microarray (Agilent, 4×44 K, G4131F) using an Agilent Gene Expression Hybridization Kit (Agilent p/n 5188–5242) as recommended by the manufacturer. Following microarray hybridization and washing, the processed slides were scanned with an Agilent DNA microarray scanner (Agilent p/n G2565BA). The steps above were performed following the instructions of the manufacturer's protocol.

### Microarray data analyses

The raw gene expression data were extracted from Agilent Feature Extraction Software (Version 10.5.1.1) and imported into Agilent GeneSpring GX software (version11.0) for further analysis. The 30 microarray data sets were normalized in GeneSpring GX using the Agilent Feature Extraction one-color scenario (primarily using quantile normalization). All of the data were interpreted using the log-ratio setting. Differentially expressed (DE) genes were identified by fold-change, t-test and P-value screening between the two groups at each time point. The DE genes were identified using a threshold of fold-change ≥2.0 and a P-value ≤0.05. To compare gene expression profiles among samples for the same time point, unsupervised hierarchical clustering was performed based on the values of the DE genes at each post-surgical time point. Gene ontology (GO) analysis was used to associate DE genes with GO categories. The GO categories were derived from Gene Ontology (www.geneontology.org) and comprised three structured networks (biological process, cellular component and molecular function) of defined terms to describe the gene product gene ontology attributes. The Fisher P-value was used to denote the significance of the GO term enrichment in the DE gene list. The lower the p-value, the more significant the GO terms. (a *p*-value ≤0.05 was recommended). All the microarray data are MIAME compliant, and the raw data are available through the GEO database with accession number GSE30322.

### Real-time polymerase chain reaction (PCR) analysis

To quantitatively examine gene expression in subchondral bone RNA samples, ten genes involved in osteoclast, osteoblast and bone remodeling in OA obtained from the microarray analysis were selected as targets. GAPDH was used as the internal control. PCR primers (**Table 9**) were designed based on cDNA sequences from the NCBI Sequence database. Quantitative SYBR-Green real-time PCR was used for five independent RNA samples from each treatment group for each time point. Each reaction was repeated three times. A Strand cDNA Synthesis Kit (GeneCopoeia, Rockville, USA) was used to generate the first-strand cDNA from the isolated RNA. The real-time PCR reactions were prepared using All-in-One™ qPCR Mix (GeneCopoeia, Rockville, USA) and All-in-One™ qPCR Primers (GeneCopoeia, Rockville, USA) according to the manufacturer's instructions. Real-time PCR and data analyses were performed using the iQ5 Real Time PCR Detection System (Bio-Rad). The cycling conditions were optimized to 40 cycles of the following protocol: denaturation at 95ssion. Student's *t*-tests were performed to determine the statistical significance of the differences between the means of the E-Group and the S-Group at each time point. All of the data were analyzed with SPSS for Windows 19.0, and the critical value ssion. Student's *t*-tests were performed to determine the statistical significance of the differences between the means of the E-Group and the S-Group at each time point. All of the data were analyzed with SPSS for Windows 19.0, and the critical value for significance was set at P = 0.05.

**Table 9:** Primers of real-time PCR target genes and internal control GAPDH.

GenBank Accession	Symbol	F-Primer (5′-3′)	R-Primer (5′-3′)
NM_017008.3	GAPDH	CAGCCGCATCTTCTTGTGC	GGTAACCAGGCGTCCGATA
NM_133523.2	Mmp3	CGGTGGCTTCAGTACCTTTC	ACCTCCTCCCAGACCTTCA
NM_019144.1	Acp5	CACAAATTGCCTACTCCAAGATC	AGTCGTCGGAATTGCCACA
NM_057149	Tnfsf11	AGACACAGAAGCACTACCTGACTC	GGCCCCACAATGTGTTGTA
NM_013059	Alp	GACTGACCCTTCCCTCTCG	GTGGTCAATCCTGCCTCCT
NM_001108550.1	Postn	AGCATCTTCCTCAGCCTCCT	TCCCCAATCAGAATCTCCCT
NM_001082479.1	Igf1	ACACTGACATGCCCAAGACTCA	GCTCAAGCAGCAAAGGATCT
NM_053384	Ihh	ACAATCCCGACATCATCTTCA	ATCCCAGCCTTCCGTCAC
NM_021578	Tgf-β1	ATGGTGGACCGCAACAAC	ACAGCAATGGGGGTTCTG
NM_001108168	Bmp5	AGAGCAGCCAGCAAACGG	GATCGCGGAAACTCACATAGA
NM_001014008	Aspn	TGTCCAACAGTGCCAAAGATG	CCAACAACGCAGCGAAAC

GAPDH, glyceraldehyde-3-phosphate dehydrogenase; Mmp3, matrix metallopeptidase 3; Acp5, acid phosphatase 5 tartrate resistant; Tnfsf11, tumor necrosis factor (ligand) superfamily, member 11; Alp, alkaline phosphatase; Postn, periostin osteoblast specific factor; IGF1, insulin-like growth factor 1; Ihh, Indian hedgehog; Tgf-β1, transforming growth factor beta 1; Bmp5, bone morphogenetic protein 5; Aspn, asporin.

### Immunohistochemistry

To validate the microarray gene expression data at the protein level, Mmp3 and Aspn proteins were selected for immunohistochemical analyses. Following deparaffinization and hydration, 5-µm sections from the medial femoral condyle of each joint were incubated with 0.3% hydrogen peroxide for 15 min. This step was followed by incubation in 10% goat serum for 30 min at room temperature to block non-specific epitopes. The sections were then incubated with anti-Mmp3 polyclonal antibody (Abcam, Cambridge, MA) at a dilution of 1∶80 and anti-Aspn polyclonal antibody (Abcam, Cambridge, MA) at a concentration of 6 µg/ml at 4°C. A negative control was treated with PBS. The sections were incubated overnight with primary antibody, washed 3 times with PBS followed by incubation with horseradish peroxidase (HRP)-conjugated secondary antibodies at 37°C for 30 min. Subsequently, all sections were reacted with Diaminobenzidine (DAKO, USA) as an HRP substrate for 10 minutes. This process was followed counterstaining with Mayer's hematoxylin (Sigma Corp., St. Louis, MO). The sections used for the analysis of each protein were obtained from 5 different animals with reproducible results. The images were captured by a Leica DMI 3000 B microscope (Germany) and software (Leica Application Suite V3).

## Supporting Information

Table S1 Supporting table.
(XLS)

Table S2 Representative annotated genes that are dysregulated at the three time points.
(DOC)

Table S3 Supporting table.
(XLS)
