# Supplementary material for: Gene Expression Analyses of Subchondral Bone in Early Experimental Osteoarthritis by Microarray
Source: PLoS One. 2012 Feb 27;7(2):e32356. doi: 10.1371/journal.pone.0032356 (PMC3288107; doi:10.1371/journal.pone.0032356)
Supplement: Table S2 — Representative annotated genes that are dysregulated at the three time points. (DOC) [file pone.0032356.s002.doc]

Supplementary Table 2. Representative annotated genes that are dysregulated at the three time points.

|  |  |  | **Direction of Dysregulation** | | |  |
| --- | --- | --- | --- | --- | --- | --- |
| **GeneBank** | **Symbol** | **Descrption** | **1W** | **2W** | **4W** | **Biological Process** |
| NM_001107758 | Meis2 | Meis homeobox 2 | ↑ | ↑ | ↑ | response to growth factor stimulus |
| NM_001012118 | Osr2 | odd-skipped related 2 | ↑ | ↑ | ↑ | bone morphogenesis |
|  |  |  |  |  |  | chondrocyte differentiation |
|  |  |  |  |  |  | embryonic digit morphogenesis |
|  |  |  |  |  |  | embryonic forelimb morphogenesis |
|  |  |  |  |  |  | embryonic hindlimb morphogenesis |
|  |  |  |  |  |  | embryonic leg joint morphogenesis |
|  |  |  |  |  |  | embryonic skeletal joint development |
|  |  |  |  |  |  | embryonic skeletal joint morphogenesis |
|  |  |  |  |  |  | embryonic skeletal system morphogenesis |
|  |  |  |  |  |  | osteoblast proliferation |
|  |  |  |  |  |  | positive regulation of bone mineralization |
| NM_001107787 | Pax1 | paired box 1 | ↑ | ↑ | ↑ | bone morphogenesis |
| NM_012550 | Ednra | endothelin receptor type A | ↑ | ↑ | ↑ | cellular response to mechanical stimulus |
|  |  |  |  |  |  | elevation of cytosolic calcium ion concentration |
|  |  |  |  |  |  | elevation of cytosolic calcium ion concentration involved in G-protein signaling coupled to IP3 second messenger |
|  |  |  |  |  |  | negative regulation of apoptosis |
|  |  |  |  |  |  | positive regulation of calcium ion transport |
|  |  |  |  |  |  | positive regulation of cell proliferation |
|  |  |  |  |  |  | positive regulation of inflammatory response |
|  |  |  |  |  |  | positive regulation of protein phosphorylation |
|  |  |  |  |  |  | positive regulation of release of sequestered calcium ion into cytosol |
|  |  |  |  |  |  | vasoconstriction |
| NM_199502 | Chrdl1 | kohjirin | ↑ | ↑ | ↑ | cell fate determination; |
|  |  |  |  |  |  | ossification |
| NM_001107526 | Blm | Bloom syndrome homolog | ↓ | ↓ | ↓ | ATP catabolic process |
| NM_182842 | Camk1g | calcium/calmodulin-dependent protein kinase IG | ↓ | ↓ | ↓ | calcium-mediated signaling |
|  |  |  |  |  |  | protein phosphorylation |
| NM_001000435 | Olr1196 | olfactory receptor 1196 | ↓ | ↓ | ↓ | G-protein coupled receptor protein signaling pathway |
| NM_023026 | Agap2 | ArfGAP with GTPase domain, ankyrin repeat and PH domain 2 | ↓ | ↓ | ↓ | regulation of ARF GTPase activity |
|  |  |  |  |  |  | small GTPase mediated signal transduction |
| NM_001107630 | Vgll2 | vestigial like 2 | ↓ | ↓ | ↓ | positive regulation of JAK-STAT cascade |
|  |  |  |  |  |  | regulation of ARF GTPase activity |
|  |  |  |  |  |  | small GTPase mediated signal transduction |
| NM_001000016 | Olr1439 | olfactory receptor 1439 | ↓ | ↓ | ↓ | G-protein coupled receptor protein signaling pathway |
| NM_019312 | Itpkb | inositol 1,4,5-trisphosphate 3-kinase B | ↓ | ↑ | ↓ | MAPKKK cascade |
|  |  |  |  |  |  | T cell differentiation |
|  |  |  |  |  |  | cell surface receptor linked signaling pathway |
|  |  |  |  |  |  | phosphorylation |
|  |  |  |  |  |  | positive regulation of Ras protein signal transduction |
|  |  |  |  |  |  | positive regulation of alpha-beta T cell differentiation |
|  |  |  |  |  |  | positive thymic T cell selection |
|  |  |  |  |  |  | thymic T cell selection |
